# Hepatitis C virus and hepatitis B virus in indolent lymphomas: Prospective data from the international observational NF10 study

**DOI:** 10.1111/bjh.70439

**Published:** 2026-03-29

**Authors:** Luca Arcaini, Michele Merli, Sara Rattotti, Maria Elena Nizzoli, Vittoria Tarantino, Chiara Consoli, Annalisa Talami, Roberta Murru, Marina Deodato, Emanuele Cencini, Francesca Re, Carlo Visco, Guilherme Duffles, Michele Spina, Ombretta Annibali, Alessandro Pulsoni, Andrés J. M. Ferreri, Caterina Stelitano, Elsa Pennese, Marzia Varettoni, Emilia Cappello, Gianmarco Favrin, Martina La Fauci, Luigi Marcheselli, Marco Paulli, Stefano Luminari

**Affiliations:** ^1^ Department of Molecular Medicine University of Pavia Pavia Italy; ^2^ Division of Hematology Fondazione IRCCS Policlinico San Matteo Pavia Italy; ^3^ Division Hematology Fondazione IRCCS Ca' Granda, Ospedale Maggiore Policlinico Milan Italy; ^4^ Division of Hematology Azienda Unità Sanitaria Locale – IRCCS Reggio Emilia Italy; ^5^ Division of Hematology Azienda Ospedaliera Ospedali Riuniti Villa Sofia‐Cervello Palermo Italy; ^6^ Division of Hematology, Department of Molecular Biotechnologies and Health Sciences University of Torino and AOU “Città della Salute e della Scienza di Torino” Torino Italy; ^7^ Clinical and Experimental Medicine Doctorate School Università degli Studi di Modena e Reggio Emilia Italy; ^8^ Hematology and Stem Cell Transplantation Unit Ospedale Oncologico A. Businco, ARNAS “G. Brotzu” Cagliari Italy; ^9^ Division of Hematology Niguarda Cancer Center, ASST Grande Ospedale Metropolitano Niguarda Milan Italy; ^10^ Division of Hematology Azienda Ospedaliera Universitaria Senese and University of Siena Siena Italy; ^11^ Division of Hematology Azienda Ospedaliero‐Universitaria di Parma Parma Italy; ^12^ Hematology and Bone Marrow Transplant Unit, Department of Engineering for Innovative Medicine University of Verona Verona Italy; ^13^ Hematology and Hemotherapy Centre University of Campinas Sao Paulo Brazil; ^14^ Division of Medical Oncology and Immune‐Related Tumors Centro di Riferimento Oncologico di Aviano (CRO), IRCCS Aviano Italy; ^15^ Division of Hematology, Stem Cell Transplantation University Campus Bio‐Medico Rome Italy; ^16^ Hematology, Sapienza University – Polo Pontino, Department of Translational and Precision Medicine S.M. Goretti Hospital Latina Italy; ^17^ Lymphoma Unit, Department of Onco‐Hematology IRCCS San Raffaele Scientific Institute Milan Italy; ^18^ Grande Ospedale Metropolitano, Bianchi Melacrino Morelli Ematologia Reggio Calabria Reggio Calabria Italy; ^19^ Department of Hematology, Lymphoma Unit Ospedale Spirito Santo Pescara Italy; ^20^ Fondazione Italiana Linfomi ETS Alessandria Italy; ^21^ Division of Pathology Fondazione IRCCS Policlinico San Matteo Pavia Italy; ^22^ Department CHIMOMO University of Modena and Reggio Emilia Modena Italy

**Keywords:** hepatis C virus, hepatitis B virus, indolent lymphoma, marginal zone lymphoma

To the Editor,

Hepatitis C virus (HCV) chronic infection has been unequivocally associated with a wide spectrum of B‐cell lymphoproliferative disorders,^1^ ranging from mixed cryoglobulinaemia to overt B‐cell non‐Hodgkin lymphomas (B‐NHL), including both indolent, especially marginal zone lymphomas (MZL), and aggressive subtypes, mainly diffuse large B‐cell lymphomas (DLBCL).^2^, ^3^ HCV has been estimated as associated with up to 20% of B‐NHL cases in Italy, 14% in Japan and 11% in the United States, while rates in the remaining European countries are lower (6%).^4^

Among indolent lymphomas, MZL are a group of indolent B‐cell non‐Hodgkin lymphomas characterized by their diverse clinical presentations and HCV‐associated MZL represents an antigen‐driven model of lymphomagenesis.^5^ However, HCV infection has also been associated with other indolent lymphomas^6^ and the prevalence of HCV infection cases varies widely across different geographical areas due to the specific epidemiology of the infection.

In the last two decades, retrospective observations reported that antiviral therapy with either interferon‐based or, more recently, interferon‐free combinations is able to induce a significant rate of lymphoma regression in HCV‐positive MZLs.^7^ A recent study from Fondazione Italiana Linfomi (FIL_BArT) prospectively investigated the use of direct‐acting antivirals (DAAs) as primary treatment in patients with HCV‐positive indolent lymphomas not requiring immediate conventional therapy.^8^

Hepatitis B virus (HBV) infection has been increasingly linked to various types of malignancies, including lymphomas.^9^ The association between HBV infection and NHL has been demonstrated for (Hepatitis B surface Antigen) HbsAg‐positive patients, although patients with occult HBV infection have been shown to also be at high risk of NHL.^10^ Meta‐analyses confirmed this association, regardless of the region and endemicity of HBV,^11^ even if only a few studies have analysed the characteristics of HBV‐associated NHL in low‐endemicity regions.^12^

The relationship between HBV and lymphoma is further complicated by the impact of immunosuppressive treatments, which are commonly used in the management of lymphoma. Immunosuppressive therapies, such as chemotherapy and monoclonal antibodies, can lead to viral reactivation in HBV carriers, resulting in acute liver failure and other serious complications.^13^ In patients with lymphoma and active HBV, antiviral treatment has been shown to reduce the risk of reactivation and improve outcomes.

While previous studies have identified various prognostic factors, the influence of chronic infections such as HCV and HBV on disease progression remains poorly understood.

This study aimed to evaluate the prevalence of HCV and HBV in MZL patients enrolled in the NF10 study (NCT02904577) and determine their impact on outcome (additional information reported in Material S1).

Of 1340 patients enrolled, HCV serology was available for 1267 of which 768 MZLs (61%), divided into 327 extra‐nodal MZL (43%), 255 splenic MZL (33%), 81 nodal MZL (11%) and 105 disseminated MZL (14%), and was positive in 6.6% ranging from 2.3% in small lymphocytic lymphoma (SLL) to 12.4% in nodal marginal zone lymphoma (NMZL) and in disseminated MZL.

Forty‐three per cent of patients in the cohort had age ≥70 years, 46% were females and 82% presented with stage III–IV. Age ≥ 70 years, female sex, absolute lymphocyte count (ALC) <1 × 10^9^/L, MZL histology and early years of registration (2010–2016) were associated with higher rates of positive HCV serology (Table 1). In multivariate logistic analysis, age and female sex were independently correlated with HCV infection, while MZL subtypes showed a worthy of note association with the positivity rate versus non‐MZLs (Table S1).

**TABLE 1:** Patients characteristic at diagnosis with positivity rate of hepatitis C virus (HCV)with available serology (percentage by rows).

Parameter	HCV, *n* (%)		*p*‐value	Total
	Negative	Positive		*n* (%)
*Age*				
<70	691 (95)	37 (5)		728 (57)
≥70	492 (91)	47 (9)	0.012	539 (43)
*Sex*				
Male	649 (95)	31 (5)		680 (54)
Female	534 (91)	53 (9)	0.001	587 (46)
*Stage*				
I–II	210 (94)	14 (6)		224 (18)
III–IV	947 (93)	59 (7)	0.883	1016 (82)
Missing	26	1		27
*Performance status*				
0–1	1108 (94)	76 (6)		1184 (94)
>1	68 (89)	8 (11)	0.157	76 (6)
Missing	7	—		7
*Extra‐nodal sites*				
0–1	1024 (94)	68 (6)		1092 (88)
>1	133 (90)	15 (10)	0.080	148 (12)
Missing	26	1		27
*Symptoms*				
A	1033 (93)	78 (7)		1111 (88)
B	143 (96)	6 (4)	0.220	149 (12)
Missing	7	—		7

Abbreviation: HCV, hepatitis C virus.

After a median follow‐up of 56 months (range 1–114 months), the 5‐year progression‐free survival (PFS) (*n* = 327 failures) for the entire series was 71% (95% confidence interval [CI] 68–74%) for HCV− and 66% (95% CI 53–76) for HCV+ (hazard ratio [HR] 1.11, 95% CI 0.73–1.68, *p* = 0.621). The 5‐year OS (*n* = 164 deaths) for the entire cohort was 86% (95% CI 74%–88%) and did not show a difference by HCV status (log‐rank test, *p* = 0.525) (Figure 1A).

**FIGURE 1:**
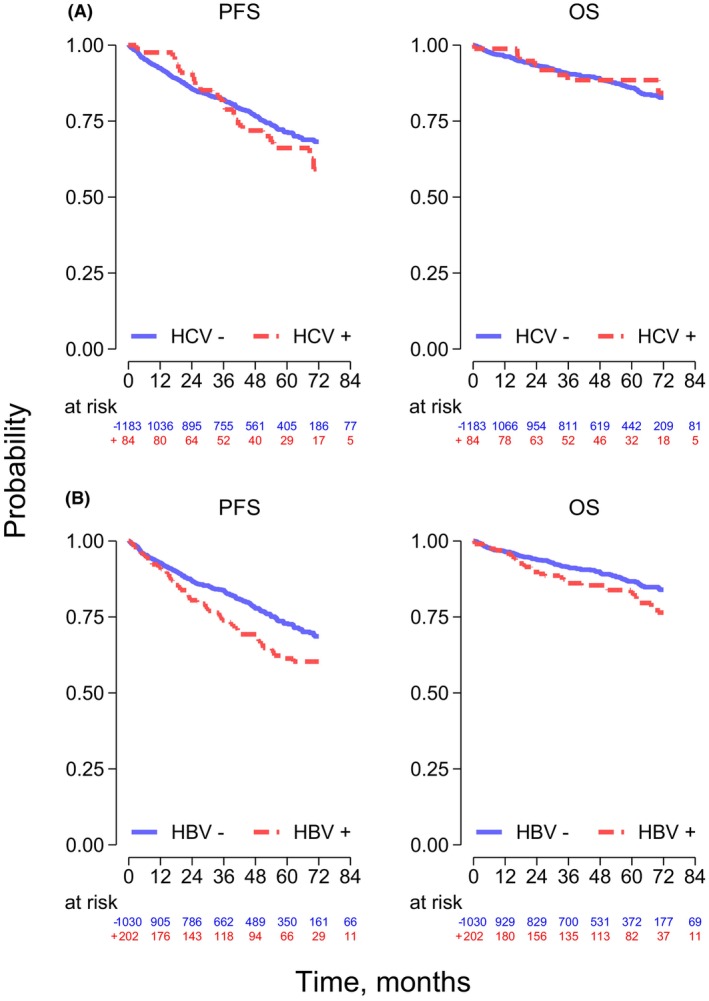
(A) Progression‐free survival and overall survival by hepatitis C virus (HCV) status in a series of 1267 patients with indolent non‐follicular lymphoma. (B) Progression‐free survival and overall survival by hepatitis B virus (HBV) status in a series of 1232 patients with indolent non‐follicular lymphoma.

HBV status was available in 1232 cases and was considered positive if HBsAg+ or HBcAb+. MZLs were 752 (61%): 320 extranodal marginal zone lymphoma, 250 splenic marginal zone lymphoma (SMZL), 81 NMZL and 101 disseminated MZL. Overall, HBV positivity was detected in 16.4% (2.2% HBsAg+ and 14.2% HBcAb+), ranging from 10% in CD5‐ NHL NOS to 21% in SMZL. Patients positive both for HBV and HCV were 26 (2%). Patients' characteristics according to HBV status were reported in Table S2.

In univariable analysis, lactate dehydrogenase (LDH) > upper normal limit (UNL), haemoglobin (Hb) < 12 g/dL and need for treatment and early years of registration (2010‐16) were associated with higher HBV+ rates; MZL histology and age were of borderline significance (Table S2).

Considering HBV positivity divided into HbsAg+ and HBcAb+, in multivariable multinomial logistic regression analysis, the relative risk ratio (RRR) of HBsAg+ compared with HBV− cases was higher for age < 70 years, males, LDH > UNL and MZL histology. Instead, considering HBcAb+, in multivariable analysis, RRR was significant only for Hb < 12 g/dL and noteworthy for sex and MZL histology (Table S3). After a median follow‐up of 56 months (range 1–114), 5‐year PFS (*n* = 319 failures) was 71% (95% CI 68%–74%) for entire series and was 73% (95% CI 70%–76%) for HBV‐ and 61% (95% CI 53%–68%) for HBV+ (HR 1.42, 95% CI 1.09–1.86, *p* = 0.010) (Figure 1B). The PFS was 59% for HBsAg+ and 62% for HBcAb+ (*p* = 0.952). In multivariable Cox regression, considering the entire series with complete data (1126/1232, 91%), the PFS was negatively associated with HBV+, age ≥70 years, B symptoms, LDH > UNL, Eastern Cooperative Oncology Group (ECOG) performance status >1, Hb < 12 g/dL, platelets <1 × 10^9^/L and non‐MZL histology (Table S4). Thus, adjusted by potential confounding, the HBV positivity retains its worse prognostic role.

Five‐year OS (*n* = 164 deaths) was 86% (95% CI 84%–88%) for the entire cohort: 87% (95% CI 84%–89%) for HBV− and 83% (95% CI 76%–88%) for HBV+ cases (HR 1.58, 95% CI 1.10–2.26, *p* = 0.013) (79% for HBsAg+ and 83% for HBcAb+ cases, *p* = 0.772). The cause of death was disease progression in 33% of HBV+ and 32% of HBV−. Adjusted in a multivariable Cox model for the same covariates selected in PFS, the HR for HBV+ was 1.43 (95% CI 0.98–2.07, *p* = 0.060): Although weakened, HBV+ showed a potential prognostic role also in OS.

Our findings provide a comprehensive evaluation of the impact of chronic hepatitis infections on indolent lymphomas, particularly MZL, within the NF10 study. The results confirm the established association between HCV and MZL but, notably, do not suggest a significant effect of HCV positivity on overall survival (OS) or PFS. In contrast, HBV positivity emerged as a critical prognostic factor, with a statistically significant negative impact on both PFS and OS.

The observed higher prevalence of HCV positivity in nodal and disseminated MZL subtypes supports the hypothesis of an antigen‐driven mechanism in lymphomagenesis. Chronic antigenic stimulation by HCV has long been considered a key driver in MZL pathogenesis, as suggested by previous studies demonstrating lymphoma regression following antiviral treatment in HCV‐positive cases.^8^ However, our data indicate that, while HCV infection remains relatively frequent among MZL patients, its influence on long‐term outcomes may be limited in the current era, potentially due to improved antiviral therapies. The declining prevalence of HCV positivity in patients recruited in later years of the study suggests an overall reduction in HCV burden, likely reflecting public health initiatives, enhanced screening and the widespread availability of DAAs.^1^ This decline underscores the evolving epidemiology of HCV‐associated lymphomas and highlights the importance of continuous surveillance.

In contrast, HBV positivity was associated with significantly worse outcomes, with HBV‐positive patients experiencing a lower 5‐year PFS (61% vs. 73%) and OS (83% vs. 87%) compared to HBV‐negative counterparts. Notably, this negative impact persisted even when stratifying for HBsAg‐positive and HBcAb‐positive patients, suggesting that both active and occult HBV infections contribute to adverse clinical outcomes.

HBV‐associated DLBCL develops through a multistep process driven by multiple pathways within an inflammatory and immunosuppressive microenvironment that promotes progression. Moreover, the risk of HBV reactivation during immunosuppressive treatment remains a significant concern, often requiring antiviral prophylaxis.^14^

Our data emphasize the need for stringent HBV screening and prophylactic antiviral strategies in HBV‐positive patients undergoing systemic therapy for lymphoma.

Another notable finding is the higher HBV positivity rate in patients enrolled in the earlier phase of the study, suggesting a potential decline in HBV prevalence over time. This trend could be attributed to widespread HBV vaccination programmes and better management of HBV carriers.^15^ However, given the significant impact of HBV positivity on clinical outcomes, continued vigilance is warranted.

From a clinical perspective, our results advocate for the routine inclusion of HBV status in the risk stratification of MZL patients. While HCV positivity alone does not appear to justify a different treatment approach, HBV‐positive patients may benefit from more aggressive monitoring and tailored management strategies. Future research should focus on elucidating the biological mechanisms underlying the negative prognostic impact of HBV and evaluating potential therapeutic interventions to mitigate its effects.

Overall, this study provides crucial prospective data on the interplay between chronic hepatitis infections and indolent lymphomas. While HCV prevalence appears to be declining, HBV remains an important adverse prognostic factor. These findings reinforce the need for personalized treatment approaches based on viral status and highlight the ongoing impact of chronic viral infections on lymphoma outcomes.

## AUTHOR CONTRIBUTIONS

**Luca Arcaini:** Data curation; formal analysis; investigation; methodology; project administration; resources; supervision; visualization; writing – original draft; writing – review and editing. **Michele Merli:** Data curation; resources; writing – original draft; writing – review and editing. **Sara Rattotti:** Data curation; resources; writing – review and editing. **Maria Elena Nizzoli:** Data curation; resources; writing – review and editing. **Vittoria Tarantino:** Data curation; resources; writing – review and editing. **Chiara Consoli:** Data curation; resources; writing – review and editing. **Annalisa Talami:** Data curation; resources; writing – review and editing. **Roberta Murru:** Data curation; resources; writing – review and editing. **Marina Deodato:** Data curation; resources; writing – review and editing. **Emanuele Cencini:** Data curation; resources; writing – review and editing. **Francesca Re:** Data curation; resources; writing – review and editing. **Carlo Visco:** Data curation; resources; writing – review and editing. **Guilherme Duffles:** Data curation; resources; writing – review and editing. **Michele Spina:** Data curation; resources; writing – review and editing. **Ombretta Annibali:** Data curation; resources; writing – review and editing. **Alessandro Pulsoni:** Data curation; resources; writing – review and editing. **Andrés J. M. Ferreri:** Data curation; resources; writing – review and editing. **Caterina Stelitano:** Data curation; resources; writing – review and editing. **Elsa Pennese:** Data curation; resources; writing – review and editing. **Marzia Varettoni:** Data curation; resources; writing – review and editing. **Emilia Cappello:** Data curation; resources; writing – review and editing. **Gianmarco Favrin:** Data curation; resources; writing – review and editing. **Martina La Fauci:** Data curation; project administration; writing – review and editing. **Luigi Marcheselli:** Data curation; formal analysis; methodology; supervision; visualization; writing – original draft; writing – review and editing. **Marco Paulli:** Data curation; resources; writing – review and editing. **Stefano Luminari:** Data curation; formal analysis; investigation; methodology; project administration; resources; supervision; visualization; writing – original draft; writing – review and editing.

## FUNDING INFORMATION

The authors declare no sources of funding to perform this retro‐spective study, which is not a clinical trial.

## CONFLICT OF INTEREST STATEMENT

The authors declare no conflicting financial interest with the sub‐mission of this article. L.A. EUSA Pharma, Novartis, Kite, Beigene, Abbvie (Speaker Bureau); Roche, Janssen‐Cilag, Verastem, Incyte, EUSA Pharma, Celgene/Bristol Myers Squibb, Kite/Gilead, ADC Therapeutics, Novartis (Participation on a Data Safety Monitoring Board or Advisory Board); Roche, AstraZeneca (Support for attending meetings and/or travel) C.A. Consultancy or speaker bureau: AbbVie, BMS, Astra Zeneca, Servier, Incyte, Roche, Pfizer, Novartis, Gentili, Janssen, Kite‐Gilead, BeOne, Lilly, Kyowa Kirin.

## ETHICS APPROVAL STATEMENT

This study was approved by the local ethical committee and respects the principles of the Declaration of Helsinki.

## Supporting information


Table S1.

## Data Availability

The data that support the findings of this study are available on request from the corresponding authors. The data are not publicly available due to privacy or ethical restrictions.
