# Supplementary material for: Hepatitis C virus and hepatitis B virus in indolent lymphomas: Prospective data from the international observational NF10 study
Source: Br J Haematol. 2026 Mar 29;208(6):2229–34. doi: 10.1111/bjh.70439 (PMC13267467; doi:10.1111/bjh.70439)
Supplement: Supplementary file 1 — Table S1. [file BJH-208-2229-s001.docx]

**NF10 study**

NF10 is a prospective observational study of 1340 newly diagnosed indolent non-follicular lymphoma (INFL) patients consecutively enrolled from July 2010 through May 2019 by 65 centers in Europe and South America. Serologic testing was performed for HCV and HBV. For all eligible patients, clinical and laboratory data were collected at the time of starting systemic treatment, which was considered as the index date for all subsequent study evaluations. Histologic diagnosis was required on tissue or bone marrow biopsy and was based on local assessment.

The study was conducted in accordance with the Declaration of Helsinki Ethical Principles and Good Clinical Practices and was approved at each site by an ethics committee. Signed consent form was mandatory for all enrolled patients.

Baseline variables were summarized as absolute and percentage frequencies, with continuous covariates dichotomized according to literature data. Association between covariates and HCV+ or HBV+ was evaluated by means of Fisher’s exact probability or Chi2 test, the effect of covariate on marker was estimated by logistic regression and expressed as odds ratio (OR) with 95% confidence interval (95%CI), either in univariable or multivariable analysis. Since HBV positivity was split in HBcAb+ and HbsAg+, a multinomial logistic regression was performed and the association of covariates on HBV status was estimated as relative-risk ratios (RRR) with 95%CI.

The impact of viral status on progression-free survival (PFS) and overall survival (OS) was assessed using Kaplan-Meier analysis and Cox regression models.

PFS was calculated from the time of systemic treatment start to the date of subsequent progression, or death due to any cause. The secondary endpoint was OS, which was defined from the date of systemic treatment to the date of death for any cause. Patients without events for both PFS and OS were censored at the time of last follow-up.^1^ PFS and OS were estimated by the method of Kaplan-Meier. The log-rank test was used to compare different groups and hazard ratios (HR) with 95%CI were estimated from Cox proportional hazard (PH) regression^2^ either in univariable or multivariable models. PH was check by the Grambsch and Therneau test.

1. Cheson BD, Fisher RI, Barrington SF, et al. Recommendations for initial evaluation, staging, and response assessment of hodgkin and non-hodgkin lymphoma: The lugano classification. *Journal of Clinical Oncology*. Preprint posted online 2014. doi:10.1200/JCO.2013.54.8800

2. Cox D. Regression models and life tables. *J R Stat Soc*. 1972;34:187-220.

**Supplemental Table 1 -** Multivariable logistic regression on HCV positivity by patient’s characteristics (cohort n=1196)

| **Parameter** |  | **OR (95%CI)** | **p-value** |
| --- | --- | --- | --- |
| **Age** | ≥ 70 | 1.75 (1.10-2.77) | 0.018 |
| **Sex** | Female | 2.05 (1.28-3.28) | 0.003 |
| **Extranodal sites** | > 1 | 1.51 (0.83-2.77) | 0.180 |
| **Lymphocyte count** | < 1 x 10^9^/L | 1.67 (0.94-2.95) | 0.078 |
| **Symptoms** | B | 0.49 (0.21-1.16) | 0.106 |
| **Histology** | MZL | 1.61 (0.96-2.70) | 0.073 |
|  |  |  |  |
| ***H-L test **** |  |  | *0.856* |
| ***AUC-ROC curve*** |  | *0.662* |  |

* Hosmer-Lemeshow goodness-of-fit test

**Supplemental Table 2 -** Patients characteristic at diagnosis with available HBV marker data (percentage by rows).

| **Parameter** |  | **HBV, n (%)** | | | **p-value** | **Total** |
| --- | --- | --- | --- | --- | --- | --- |
|  |  | **Negative** | **HBsAg+** | **HBcAb+** |  | **n (%)** |
| **Age** | < 70 | 587 (83) | 21 (3) | 99 (14) |  | 707 (57) |
|  | ≥ 70 | 443 (84) | 6 (1) | 76 (14) | 0.095 | 525 (43) |
| **Sex** | Male | 541 (82) | 19 (3) | 99 (15) |  | 659 (53) |
|  | Female | 489 (85) | 8 (1) | 76 (13) | 0.128 | 573 (47) |
| **Stage** | I-II | 192(86) | 2 (1) | 28 (13) |  | 222 (18) |
|  | III-IV | 814 (83) | 25 (2) | 144 (15) | 0.247 | 983 (82) |
| ***Missing*** |  | *24* | *-* | *3* |  | *27* |
| **Performance status** | 0-1 | 968 (84) | 25 (2) | 156 (14) |  | 1149 (94) |
|  | > 1 | 56 (74) | 2 (3) | 18(24) | 0.043 | 76 (6) |
| ***Missing*** |  |  |  |  |  |  |
| **Extranodal sites** | 0-1 | 890 (84) | 22 (2) | 148 (14) |  | 1060 (88) |
|  | > 1 | 116 (80) | 5 (3) | 24 (17) | 0.317 | 145 (12) |
| ***Missing*** |  | *24* | *-* | *3* |  | *27* |
| **Symptoms** | A | 904 (84) | 20 (2) | 154 (14) |  | 1078 (88) |
|  | B | 120 (82) | 7 (5) | 20 (14) | 0.103 | 147 (12) |
| ***Missing*** |  | *6* | *-* | *1* |  | *7* |
| **LDH** | ≤ ULN | 754 (85) | 14 (1) | 123 (14) |  | 1. 5) |
|  | > ULN | 231 (79) | 11 (4) | 50 (17) | 0.026 | 292 (25) |
| ***Missing*** |  | *45* | *2* | *2* |  | *49* |
| **Lymphocytes count** | ≥ 1 x 10^9^/L | 864 (84) | 20 (2) | 147 (14) |  | 1031 (86) |
|  | < 1 x 10^9^/L | 131 (81) | 5 (3) | 26 (16) | 0.454 | 162 (14) |
| ***Missing*** |  | *35* | *2* | *2* |  | *39* |
| **Hemoglobin** | ≥ 12 mg/dL | 668 (86) | 14 (2) | 92 (12) |  | 774 (63) |
|  | < 12 mg/dL | 357 (79) | 13 (3) | 83 (18) | 0.003 | 453 (37) |
| ***Missing*** |  | *5* | *-* | *-* |  | *5* |
| **Platelets count** | ≥ 100 x 10^9^/L | 922 (84) | 23 (2) | 158 (14) |  | 1103 (90) |
|  | < 100 x 10^9^/L | 103 (83) | 4 (3) | 17 (14) | 0.661 | 124 (10) |
| ***Missing*** |  | *5* | *-* | *-* |  | *5* |
| **Histology** | ENMZL | 270 (26) | 6 (22) | 44 (25) | 0.450 | 320 (26) |
|  | SMZL | 196 (19) | 8 (30) | 46 (26) |  | 250 (20) |
|  | SLL | 102 (10) | 1 (4) | 20 (11) |  | 123 (10) |
|  | LPL | 239 (23) | 5 (19) | 33 (19) |  | 277 (22) |
|  | CD5- | 72 (7) | 1 (4) | 7 (4) |  | 80 (6) |
|  | NMZL | 68 (7) | 3 (11) | 10 (6) |  | 81 (7) |
|  | Diff-MZL | 83 (8) | 3 (11) | 15 (9) |  | 101 (8) |
| **Approach** | Treated | 507 (80) | 17 (3) | 109 (17) |  | 633 (51) |
|  | W&W | 386 (88) | 8 (2) | 44 (10) |  | 438 (36) |
|  | Treated W&W* | 137 (85) | 2 (1) | 22 (14) | 0.010 | 161 (13) |
| **Year diagnosis** | 2010/13 | 349 (80) | 15 (3) | 70 (16) |  | 434 (35) |
|  | 2014/16 | 473 (84) | 7 (1) | 82 (15) |  | 562 (46) |
|  | 2017/18 | 208 (88) | 5 (2) | 23 (10) | 0.023 | 236 (19) |

Total: percentage by column. LDH: lactate dehydrogenase, ULN: upper limit of normality; EMZL: extranodal SMZL: Splenic MZL; LPL: lymphoplasmacytic lymphoma; CD5-: CD5 negative MZL; NMZL: Nodular MZL; Diss-MZL: Disseminate MZL; marginal zone lymphomas; W&W: watch and wait. *Treat. W&W: patients treated after interruption of W&W. NOTE. Because of rounding, percentages may not total 100.

**Supplemental Table 3 -** Multivariable multinomial logistic regression on HBV positivity by patient’s characteristics (cohort n=1182)

| **Baseline outcome: HBV negative** | | |  |
| --- | --- | --- | --- |
| **HBsAg+** |  | **RRR (95%CI)** | **p-value** |
| **Age** | ≥ 70 | 0.34 (0.13-0.92) | 0.034 |
| **Sex** | Female | 0.35 (0.20-0.86) | 0.022 |
| **LDH** | > ULN | 2.45 (1.06-5.70) | 0.037 |
| **Hemoglobin** | < 12 g/dL | 1.90 (0.82-4.39) | 0.132 |
| **Histology** | MZL | 2.68 (0.98-7.33) | 0.055 |
| **HbcAb+** |  | **RRR (95%CI)** | **p-value** |
| **Age** | ≥70 | 0.97 (0.70-1.35) | 0.864 |
| **Sex** | Female | 0.75 (0.53-1.04) | 0.088 |
| **LDH** | >ULN | 1.22 (0.84-1.77) | 0.304 |
| **Hemoglobin** | < 12 g/dL | 1.68 (1.20-2.35) | 0.003 |
| **Histology** | MZL | 1.36 (0.96-1.92) | 0.083 |
|  |  |  |  |
| ***GoF*** |  |  | *0.386* |

RRR: relative risk-ratio; Hosmer-Lemeshow goodness-of-fit test; GoF: goodness of fit [Fagerland MW, Hosmer DW, Bofin AM: Multinomial goodness-of-fit tests for logistic regression models. Statistics in Medicine 27: 4238–4253; 2008]

LDH: lactate dehydrogenase, ULN: upper limit of normality; MZL: marginal zone lymphomas

**Supplemental Table 4 –** Univariable and multivariable Cox PH regression on progression-free survival for cohort with available HBV markers (n=1126, 303 failures)

| **Factor** | **Univariable** | | **Multivariable** | |
| --- | --- | --- | --- | --- |
|  | **HR (95%CI)** | **p-value** | **HR (95%CI)** | **p-value** |
| **HBV+ (HBsAg+ or HBcAb+)** | 1.44 (1.09-1.89) | 0.010 | 1.34 (1.02-1.78) | 0.038 |
| **Age ≥ 70 years** | 1.68 (1.34-2.11) | <0.001 | 1.51 (1.20-1.91) | <0.001 |
| **Female sex** | 0.95 (0.75-1.19) | 0.626 |  |  |
| **Stage III-IV** | 1.79 (1.27-2.53) | 0.001 |  |  |
| **Extranodal sites** | 0.96 (0.68-1.35) | 0.802 |  |  |
| **Symptoms B** | 1.81 (1.36-2.41) | <0.001 | 1.35 (0.99-1.83) | 0.056 |
| **LDH > ULN** | 1.78 (1.40-2.26) | <0.001 | 1.33 (1.03-1.71) | 0.027 |
| **Performance status > 1** | 2.86 (2.03-4.02) | <0.001 | 1.78 (1.24-2.58) | 0.002 |
| **Lymphocyte count < 1 x 10^9^/L** | 1.48 (1.10-1.98) | 0.009 | 1.24 (0.91-1.67) | 0.167 |
| **Platelet count < 1 x 10^9^/L** | 1.77 (1.28-2.45) | 0.001 | 1.45 (1.03-2.02) | 0.032 |
| **Hemoglobin < 12 g/dL** | 2.00 (1.60-2.51) | <0.001 | 1.26 (0.98-1.62) | 0.072 |
| **MZL’s vs non-MZL** | 0.84 (0.67-1.06) | 0.146 | 0.69 (0.54-0.88) | 0.003 |
| **Treated at diagnosis** | 1.00 |  | 1.00 |  |
| **W&W** | 0.31 (0.23-043) | <0.001 | 0.35 (0.25-0.49) | <0.001 |
| **Treated after W&W** | 1.20 (0.86-1.66) | 0.279 | 1.17 (0.83-1.63) | 0.373 |

Likelihood-ratio test final multivariable model nested in full model, p=0.444

LDH: lactate dehydrogenase, ULN: upper limit of normality; MZL: marginal zone lymphomas; W&W: watch and wait
